# MIF and CD74 as Emerging Biomarkers for Immune Checkpoint Blockade Therapy

**DOI:** 10.3390/cancers16091773

**Published:** 2024-05-04

**Authors:** Rosalyn M. Fey, Rebecca A. Nichols, Thuy T. Tran, Arthur A. Vandenbark, Rajan P. Kulkarni

**Affiliations:** 1 Department of Dermatology, Oregon Health & Science University, Portland, OR 97239, USAnicholre@ohsu.edu (R.A.N.); 2 Yale Cancer Center, Yale University School of Medicine, New Haven, CT 06510, USA; 3 Neuroimmunology Research, R&D-31, VA Portland Health Care System, Portland, OR 97239, USA; 4 Department of Neurology, Oregon Health & Science University, Portland, OR 97239, USA; 5 Department of Molecular Microbiology and Immunology, Oregon Health & Science University, Portland, OR 97239, USA; 6 Cancer Early Detection Advanced Research Center (CEDAR), Portland, OR 97239, USA; 7 Knight Cancer Institute, Oregon Health and Science University, Portland, OR 97239, USA; 8 Operative Care Division, U.S. Department of Veterans Affairs Portland Health Care System, Portland, OR 97239, USA

**Keywords:** macrophage migration inhibitory factor (MIF), CD74, biomarkers, immune-related adverse events (irAEs), immune checkpoint blockade (ICB), cancer, autoimmune disease

## Abstract

**Simple Summary:**

While immune checkpoint blockade (ICB) therapy is used successfully to treat various cancers, many patients develop immune-related adverse side effects (irAEs), which can be severe enough to cause decreases in quality of life and, in some cases, may result in treatment cessation. Here, we review current studies investigating the potential utility of the molecules MIF and CD74 as predictive biomarkers for ICB response and irAE development. We also discuss evidence for the circadian expression of MIF. Finally, we aim to highlight areas where future research will be beneficial in establishing the value of MIF and CD74 as biomarkers of ICB response.

**Abstract:**

Immune checkpoint blockade (ICB) therapy is used to treat a wide range of cancers; however, some patients are at risk of developing treatment resistance and/or immune-related adverse events (irAEs). Thus, there is a great need for the identification of reliable predictive biomarkers for response and toxicity. The cytokine MIF (macrophage migration inhibitory factor) and its cognate receptor CD74 are intimately connected with cancer progression and have previously been proposed as prognostic biomarkers for patient outcome in various cancers, including solid tumors such as malignant melanoma. Here, we assess their potential as predictive biomarkers for response to ICB therapy and irAE development. We provide a brief overview of their function and roles in the context of cancer and autoimmune disease. We also review the evidence showing that MIF and CD74 may be of use as predictive biomarkers of patient response to ICB therapy and irAE development. We also highlight that careful consideration is required when assessing the potential of serum MIF levels as a biomarker due to its reported circadian expression in human plasma. Finally, we suggest future directions for the establishment of MIF and CD74 as predictive biomarkers for ICB therapy and irAE development to guide further research in this field.

## 1. Introduction

Immune checkpoint blockade (ICB) therapy is a powerful cancer treatment that has been used with great success in a variety of cancers in recent years. Cancer cells are able to stimulate checkpoint proteins on immune cells, which impairs the immune response and promotes cancer progression; ICB drugs block the interaction between these checkpoint proteins and their ligands to enhance immune cell recognition and activation against tumor antigens, thereby driving an effective anti-tumor response [1]. In spite of its efficacy in many cases, some patients do not respond to ICB therapy (primary resistance), and some show an initial response that is lost after some time (acquired resistance) [2].

In addition, although ICB drugs are generally better-tolerated than treatments like chemotherapy, many patients treated with ICB therapy develop immune-related adverse events (irAEs) [1,3,4]. IrAEs are autoimmune-like inflammatory responses that can affect any organ system, range from mild to fatal, and may necessitate cessation of ICB therapy based on the severity [4].

Because of the potential to experience resistance or irAEs, there is great interest in discovering and characterizing biomarkers that can predict patient response to ICB therapy and irAE development. The detection and evaluation of these biomarkers is extensive and ongoing. Among the plethora of investigated biomarkers for ICB response are gut microbiome composition, checkpoint protein expression on immune or cancer cells, gene expression signatures, and tumor mutation burden [5,6,7,8]. For irAE risk, the biomarkers under investigation include autoantibodies, thyroid stimulating hormone, and the expression levels of various cytokines and chemokines [9,10,11]. However, many of these biomarkers are only predictive for certain kinds of cancers or irAEs [3]. Complicating matters further, the biomarkers used to predict ICB response are not always the same as those used to predict irAE risk. It is generally agreed that there are currently no sufficiently effective biomarkers for ICB response or irAE risk prediction. Thus, there is a need to continue the search for cancer- and irAE-specific predictors of ICB response and irAE development or to discover biomarkers that are generalizable across conditions.

The expression of the proinflammatory cytokine MIF (macrophage migration inhibitory factor) and its cognate receptor CD74 has been investigated as a prognostic indicator for various cancers, sometimes with conflicting results depending on the type of cancer and the mode of biomarker measurement (serum or tumor, mRNA or protein, etc.) [12,13,14,15,16,17,18,19,20]. In addition, MIF and CD74 have been identified as potential targets of anti-cancer therapeutics, including for cervical cancer [21], ovarian carcinoma [15], glioblastoma [22], multiple myeloma [23,24,25,26], and melanoma [12,27,28,29]. Pertinent to this area of study, multiple MIF modulators are currently being investigated as targets for anti-cancer treatment [30].

Here, we focus on the potential of MIF and CD74 as predictive biomarkers for ICB therapy, including patient response and the development of irAEs. We aim to distill and synthesize information from the literature about the roles of MIF and CD74 in cancer, to provide perspectives on these two molecules as predictive biomarkers for immune checkpoint therapy and to uncover gaps in knowledge and present them as future directions for research in the field.

## 2. A Brief Overview of MIF and CD74

As its name suggests, MIF was originally discovered as a suppressor of macrophage migration secreted by lymphocytes [30,31], but it is now known to be expressed by both lymphoid and myeloid cells in the context of innate and adaptive immunity, as well as by immune and tumor cells in the context of tumor immunity (reviewed in [32]). MIF can act to modify the action of transcription factors, enzymes, and other intracellular proteins, but it is also secreted and found in circulation as a cytokine capable of inducing inflammation [32,33].

**Figure 1:**
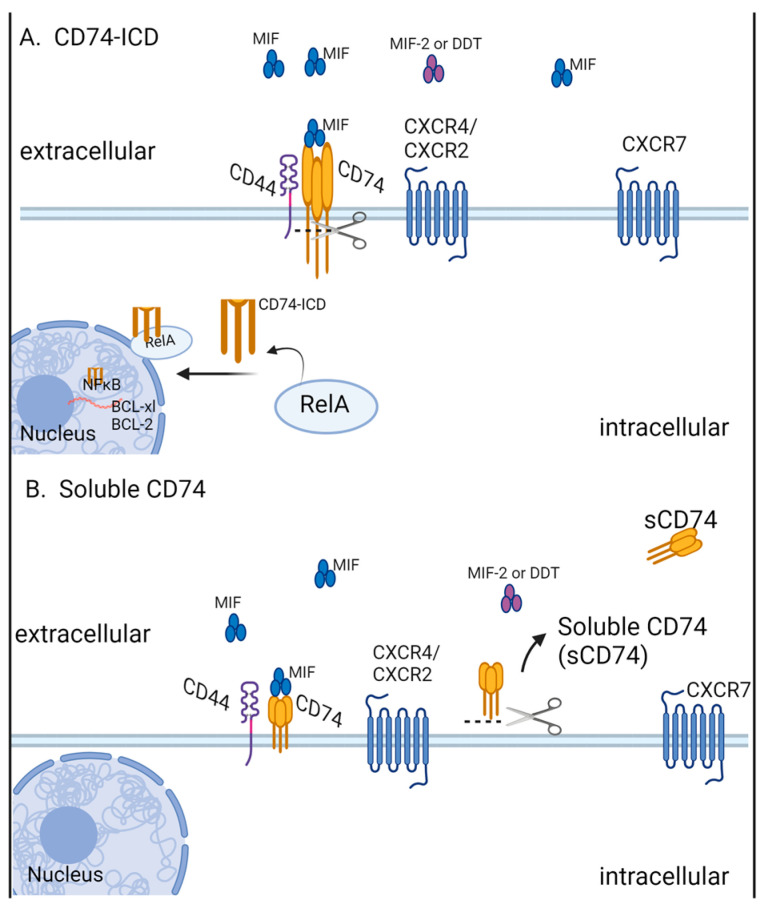
Intracellular and soluble CD74. (**A**) The release of the CD74 intercellular domain from the transmembrane portion allows CD74-ICD to exert intracellular and transcriptional effects. (**B**) Soluble CD74 is released from the cell surface via extracellular cleavage.

Although MIF was discovered in the 1960s, CD74 was not identified as its cognate receptor until 2003 [34]. CD74 is a transmembrane protein made up of extracellular, transmembrane, and intracellular domains. The intracellular domain (ICD) can be cleaved from the transmembrane portion and can act as a chaperone for the MHC class II complex [35] or enter the nucleus and activate transcriptional programs (Figure 1A) [36]. The extracellular segment of CD74 is expressed on the surface of immune and non-immune cells where it binds to MIF [34,35], which forms a cell–surface-receptor complex with CD44 [37]. This CD74/CD44 complex also binds the structurally similar MIF functional homolog D-dopachrome tautomerase (D-DT, also called MIF-2) [38,39]. As well as acting as a cell-surface MIF receptor, the extracellular portion of CD74 (soluble CD74, or sCD74) can be released from the rest of the protein by ADAM (a disintegrin and metalloproteinase) sheddases or by cysteine proteases, depending on the cell line, after which it is capable of sequestering MIF (Figure 1B) [20,34,40,41].

In addition to binding to CD74, MIF can bind to three non-cognate receptors: CXCR2, CXCR4, and CXCR7 [42,43]. CD74 has been shown to complex with CXCR2 and with CXCR4, and has important functional implications for MIF signaling [44,45]. Further, MIF-mediated signaling pathways show stronger responses when CD74 is co-expressed with CXCR2 or CXCR4 compared to either chemokine receptor alone [42]. While they are not our focus here, the roles of CXCR2, CXCR4, and CXCR7 as MIF receptors have been reviewed elsewhere [33].

The actions of MIF and CD74 are increasingly understood to be context- and cell-type-specific. They play critical roles in healthy immune cells and in the normal immune response (reviewed in [33,46,47]). For example, in addition to its immune-related function as an MHC class II chaperone, CD74 regulates transcription in healthy B cells [48]. MIF not only affects macrophage migration but has widespread functions as a signaling molecule, including being produced by T cells upon activation [49] and inhibiting neutrophil apoptosis via the action of mononuclear cells secreting CXCL8 [50].

In addition to their known effects in normal cells and systems, MIF and CD74 are increasingly recognized as playing an intricate role in cancer progression. MIF is overexpressed in various cancers, including lung cancer, osteosarcoma, metastatic melanoma, and many others [13]; CD74 expression and overexpression are also associated with diverse cancers [51]. CD74 and MIF together encourage cell proliferation and inhibit cell death [51], and MIF’s role in inflammation has been recognized as an important potential player in cancer initiation and development [52]. MIF and CD74 have been implicated in the mechanisms of the development of various cancers, including non-small-cell lung cancer [53], prostate cancer [54], head and neck squamous cell carcinoma [55], and brain tumors [56].

Further, both MIF and CD74 are important players in immune homeostasis and cancer. Immune homeostasis is a complicated balance involving many cell types throughout the body. In the context of ICB treatment, checkpoint proteins and the cells expressing them are of utmost importance in tipping this balance back toward a tumor microenvironment under immune surveillance, rather than an immunosuppressive one. It is relevant to this point that MIF has been shown to be immunosuppressive in the context of melanoma [57,58]. Cells that are important for this homeostasis include regulatory T cells, which express checkpoint proteins and act to dampen the immune response. Importantly, a recent analysis of cell-to-cell interactions in the context of lung adenocarcinoma predicted the involvement of the MIF-CD74 signaling axis in intercellular communication between regulatory T cells and other cells in the tumor microenvironment, including fibroblasts, epithelial cells, and B cells [59]. While a comprehensive discussion of MIF and CD74 in cancer is outside the scope of this work, the interested reader is referred to several excellent reviews on this topic [21,32,51,55,60].

Besides their importance in normal and malignant cell settings, MIF and CD74 are implicated in the etiology of numerous autoimmune diseases (reviewed in [46,61]), including autoimmune liver disease [41], kidney disease [62,63], and multiple sclerosis [64]. A high expression of MIF mRNA and protein in human autoimmune-associated kidney disease was described over 20 years ago by Lan et al. [65]. Both CD74 and MIF have been extensively studied in the context of rheumatoid arthritis, and MIF is now recognized as a crucial player in the development of this disease [66,67,68,69]. Recently, therapeutics aimed at disrupting MIF and/or CD74 binding have been considered in the treatment of several autoimmune diseases [64,70,71].

The well-documented involvement of MIF and CD74 in the balance of the immune system and the pathogenesis of autoimmune diseases suggests they are promising candidates for future research as biomarkers of irAE development following ICB treatment.

## 3. The Role of MIF and CD74 in the Mechanisms of Immune-Related Adverse Events (irAEs)

As ICB therapy becomes more commonly used as the first line of defense in cancer treatment, an emerging risk is the increased immune-mediated toxicity to normal tissues. These immune-related adverse events (irAEs) appear to be mediated by the overactivation of immune cells in the body due to ICB treatment, which disrupts the signaling pathways responsible for maintaining a balance of immune tolerance in the body. Various cell types are thought to be involved in this dysregulation. Immune checkpoint receptors like PD-1, PD-L1, and CTLA-4 are expressed on various T cell subsets and can inhibit effector T cells and their function. ICB drugs can reactivate these T cells, allowing the destruction of cancer cells via recognition of tumor antigens and self-antigens by blocking inhibitory signaling pathways. In addition, ICB therapy can downregulate the action of regulatory T cells (Tregs). However, this can also cause highly variable non-specific autoinflammation in non-target tissue, resulting in a strong autoimmune effect. This may be partly due to CTLA-4-expressing Tregs, which normally facilitate immunosuppression via various mechanisms, including by producing inhibitory cytokines or affecting the maturation of dendritic cells (DCs) [72]. Importantly, MIF is known to promote tumor-associated immune cell evasion by inhibiting DC migration, maturation, and antigen presentation, which helps the development of anti-tumor CD4+ and CD8+ effector T cells [32].

Activated T cells in the tumor microenvironment can express immune checkpoint molecules like PD-1 in multiple T cell differentiation states, including T follicular helper cells that reside in lymph nodes to help produce antibody-producing B cells and tissue-resident memory T cells (Trm) that remain in peripheral tissues to protect against infection or pathogens [72,73]. In a study by Reschke et al., expanded CD4+ and CD8+ Trm cells were the dominant population in biopsies of inflammatory reactions with irAE-dermatitis and irAE-colitis after checkpoint inhibitor immunotherapy; in these Trm cells, INF-γ and TNF-a were expressed along with genes like HLA-DRA, CD74, and GBP5, indicating a Th1/Tc1 phenotype [73]. In psoriasis control samples, Trm cells showed a more Th17/Tc17 polarized phenotype with upregulation in the expression of PD-1, CTLA-4, LAG-3, and other inhibitory receptors [73].

Stojanović et al. first demonstrated MIF’s ability to act upstream and influence the increase of IL-17 on a heterogeneous population of murine lymphocytes [74]. This idea of MIF-dependent regulation of Th17 cells was furthered by examining tumor-derived MIF expression in nasopharyngeal carcinoma; MIF involvement in the development, recruitment, and migration of intertumoral Th17 cells was mainly dependent on the mTOR pathway and the interaction of MIF with CXCR4 and was associated with a more favorable outcome in patients [75].

MIF also influences the B cell activation pathway at various stages of development and maturation [32]. This influence on B cell proliferation, class switching, and cytokine production is in agreement with an important newly discovered role for B cells in ICB therapy [32]. Thibult et al. demonstrated that the blockade of PD-1 pathways increased the proliferation and activation of B cells, resulting in the production of inflammatory cytokines; therefore, PD-1 plays a negative role in regulating and differentiating B cells [76].

Certain B cell subsets can regulate T cell immune responses and are termed regulatory B cells (Breg). The majority of Breg cells are identified by IL-10 production and are known to suppress allergy and autoimmunity [77,78]. Like Tregs, Breg cells can inhibit immune responses and maintain immune homeostasis and appear to have Treg-like mechanisms for induction and maintenance that can suppress pro-inflammatory cells, including monocytes, DCs, various CD4+ T helper (Th) cell subsets, and CD8+ T cells [79]. Furthermore, Bregs can directly inhibit CD4+ Th cells and CD8+ T cells via the PD-1/PD-L1 [77] and CTLA-4 pathways [80]. B cells and associated tertiary lymphoid structures are ectopic lymphoid structures that form in continual exposure to antigens and have been shown to be correlated with responses to ICB and better survival outcomes in cancer patients [78]. Activated B cells can recruit Th2 cells that produce cytokines such as IL-4, which can induce a B cell into becoming a cytokine-producing B-cell (termed B effector cells); IL-4 can also stimulate B cell expression in MHC class II, which can act as regulatory/inhibitory cell of the immune system [81]. In melanoma patients treated with combination ICB therapy, changes in B cell populations correlate with an increase in the likelihood of irAEs [72,82].

A link between the MIF-CD74 axis and the mechanisms of irAE development can also be glimpsed in antigen-presenting cells (APCs). Since, among immune cells, CD74 is mainly expressed on APCs like DCs and macrophages and functions as an MHC class II chaperone, it is critical for the antigen-specific immune response [48].

Tumor-associated macrophages (TAMs) are common cells in melanoma and are inversely correlated with patient outcome [83,84]. Depending on their maturation state, they can be both immunosuppressive and immunogenic [27]. MIF can be secreted as an immunosuppressive factor in the tumor microenvironment, and blocking CD74/MIF signaling enhances CD8+ T cell infiltration and drives macrophage pro-inflammatory conversions and anti-tumor function. Potentially using combination therapy with both anti-CTLA-4 and a MIF inhibitor could increase immune cell infiltration in the tumor and reduce the expression of PD-L1 in melanoma cells [12]. TAM infiltration is associated with poor prognosis and tumor progression in several cancers, including melanoma and pancreatic, breast, and bladder cancers, and single-cell RNA sequencing has revealed that high TAM infiltration is associated with an increase in CD8+ T cell exhaustion and the number of Tregs [85].

## 4. MIF and CD74 Predict ICB Treatment Response in Multiple Cancers

Due to their roles in cancer initiation and progression and intracellular communication in the tumor microenvironment, CD74 and MIF have been considered widely as prognostic biomarkers for various cancers, with various confidence levels [12,13,14,15,16,17,18,19,20]. More recently, with the advent of ICB therapy, both CD74 and MIF are also noted as prospective predictive biomarkers for ICB treatment response.

MIF has been investigated as a biomarker of patient response to ICB therapy in only a handful of studies to date. It has been shown that lower baseline levels of MIF protein expression in both serum and in the tumor microenvironment are correlated with a higher efficacy of anti-PD-1 ICB therapy when used in conjunction with chemotherapy as a neoadjuvant treatment for esophageal squamous cell carcinoma [86]. In non-small-cell lung cancer, a higher baseline serum MIF protein level is a weak prognostic marker of worse progression-free survival after anti-PD-1 ICB treatment but does not predict overall survival [87], and a similar trend has been shown in melanoma [12], corroborating the inverse correlation between MIF expression and patient response to ICB therapy. In addition to the research directly investigating MIF as a biomarker, recent studies have suggested a potential mechanistic role for MIF in ICB treatment response via monocytes in gastric cancer [88] and through metabolic reprogramming in a breast cancer stem cell line [89]. Future work should elucidate the cell-type-specific mechanisms of MIF in tumor control and patient response to ICB therapy.

CD74 has also been proposed as a biomarker for ICB treatment response. While high tumoral CD74 gene expression has been shown to be predictive of better overall survival in patients with cutaneous melanoma, it has also been associated with worse ICB response [90]; in this study, these two outcomes were examined independently from each other [90]. However, in a study of patients with solid tumors, increased tumoral gene expression of CD74 at baseline was associated with better overall survival after treatment with dual ICB therapy (a single agent targeting both CTLA-4 and PD-1) [91]. Interestingly, CD74 copy-number losses have been associated with a lack of response to combination anti-CTLA-4/anti-PD-1 ICB treatment in patients with advanced melanoma [92].

We note that the significance of the predictive utility of MIF/CD74 levels is dependent on the definition of ICB response measurement (progression-free survival, overall survival, etc.). Similarly, the modality chosen for MIF/CD74 measurements (serum levels, tumoral protein or gene expression levels, copy-number loss, etc.) affects the significance of the association with ICB response. Thus, while both MIF and CD74 show promise as predictive biomarkers for patient response to ICB therapy in multiple cancers, more work is needed to establish their reliability and generalizability across cancers, ICB agents, ICB response measurement, and measurements of MIF/CD74.

## 5. MIF and CD74 as Putative Predictive Biomarkers of irAE Development

In addition to playing a role in the development of auto-immune diseases, CD74 has been implicated in the mechanism and development of irAEs. In a study of patients with various solid tumors (including renal cell carcinoma) who received combination anti-CTLA-4 and anti-PD-1 ICB treatment, a higher expression of CD74 autoantibodies measured in the serum before ICB therapy was correlated with the development of irAE pneumonitis [93]. Furthermore, CD74 protein was expressed at much higher levels in the lungs of patients with ICB-induced pneumonitis compared to normal lung tissue, suggesting that the high expression of CD74 autoantibodies may have relevance in the mechanism of ICB-induced pneumonitis [93]. A follow-up study investigated CD74 autoantibody levels in the plasma of Japanese patients with renal cell carcinoma receiving ICB combination or monotherapy; in this work, CD74 autoantibody levels did not represent a predictive biomarker of pneumonitis irAE or higher-grade irAEs [94]. These conflicting reports suggest that further work is needed to establish whether CD74 can be a useful biomarker for irAE development.

While MIF has not yet been investigated as a biomarker for irAE development in humans to the best of our knowledge, studies in mice have predicted MIF-mediated cross-talk between T cells and macrophages in the context of irAEs [95,96], warranting its future exploration in studies using human tissues.

## 6. Serum MIF Expression Exhibits a Distinct Circadian Rhythm

**Figure 2:**
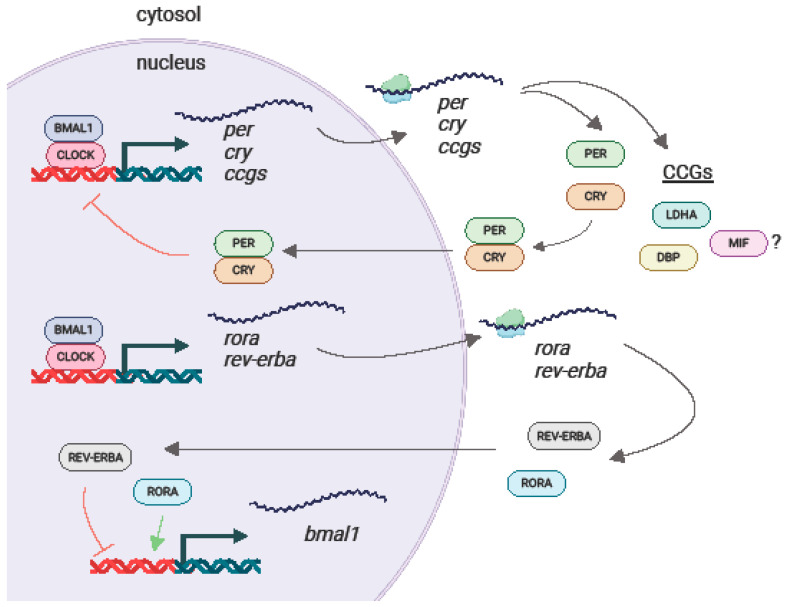
Simplified schematic of the mammalian molecular circadian clock. A CLOCK and BMAL1 heterodimer binds to the promoter regions of CRY and PER genes, activating their transcription. CRY and PER mRNA are transported out of the nucleus, where they are transcribed into protein. These two proteins form a heterodimer and translocate to the nucleus, where they repress their own transcription. CLOCK/BMAL1 also activates the transcription of RORA and REV-ERB mRNA, forming a secondary transcription/translation feedback loop that controls the expression of BMAL1. In addition to regulating the transcription of core clock genes, the CLOCK/BMAL1 heterodimer activates the rhythmic transcription of clock-controlled genes (CCGs).

Circadian rhythms are circa 24 h oscillations in behavior and physiology that are hypothesized to confer a survival advantage by allowing organisms to anticipate daily environmental changes. On the molecular level, circadian oscillations in gene expression are governed by a master circadian clock in the suprachiasmatic nucleus in mammals, where core transcription factors undergo transcription, translation, phosphorylation, and degradation over the course of approximately 24 h, regulating the transcription of thousands of clock-controlled genes (CCGs) (Figure 2) [97,98].

MIF has been shown to oscillate with 24 h frequency in at least two studies, in which MIF cytokine levels in the plasma of healthy adults showed a 24 h rhythm and peaked in the late morning [99] or the early morning [100]. Various other studies report circadian association, even when MIF oscillations are not directly measured. Night-shift workers have higher serum MIF levels compared to day-shift workers [101], indicating that circadian disruption affects MIF expression. In addition, MIF transcription is regulated by ICBP90 (also called UHRF1) [102]; UHRF1 has been shown to be a downstream effector of BMAL1, one of the core circadian clock transcription factors [103], suggesting a potential mechanism for the circadian regulation of MIF expression.

In addition, and especially important in the context of ICB treatment, MIF is intimately connected with glucocorticoids, which are widely used to treat irAEs [104,105]. Proinflammatory MIF interferes with the immunosuppressive action of glucocorticoids [46], and glucocorticoids induce MIF mRNA and protein expression [106]. Glucocorticoids oscillate with 24 h frequency, have long been known to be involved in the circadian clock as both a regulatory input and output, and play an important role in synchronizing the central and peripheral circadian clocks [107,108].

This evidence for MIF circadian expression in the serum of healthy adults, its regulation by the circadian clock, and its relationship to glucocorticoids highlight the need for careful consideration of MIF rhythmic expression patterns as the potential of MIF as a biomarker of irAE development and ICB therapy response is evaluated. To our knowledge, CD74 does not exhibit a circadian response and may therefore be a more universal biomarker, though this needs to be further investigated.

## 7. Future Directions

Although there is ample evidence that CD74 and MIF are instrumental in the development of autoimmune disorders and some indication that they may be involved in the mechanisms of irAE development, there are a limited number of studies into their utility as predictive biomarkers of irAE development after ICB treatment. CD74 has been investigated as a biomarker of pneumonitis in renal cell carcinoma, but future work is needed to examine its potential as a biomarker of other irAEs and in other cancers. To the best of our knowledge, MIF has been investigated in the context of irAE biomarkers only in mice; however, its involvement in autoimmune diseases suggests that studies in humans are warranted.

Similarly, more work is needed to examine MIF and CD74 as biomarkers of patient response to ICB therapy. Many studies have investigated CD74 as a biomarker of ICB response in melanoma; additionally, it shows promise for other cancers, including triple-negative breast cancer [17], although there is a paucity of studies exploring its utility in these cases. Although the predictive power of MIF has only been explored in a few studies, further work will establish whether it is of use generally as a predictive biomarker for ICB treatment response.

In order to use any predictive biomarker with the highest level of confidence, it is ideal to understand its circadian expression. MIF cytokine levels in the serum of healthy adults fluctuate with 24 h rhythmicity, but conflicting reports exist in the literature as to its peak expression times (early morning versus late morning). Further work is needed to establish a conclusive circadian expression profile for comparison purposes before serum MIF can be used with confidence as a predictive biomarker. Alternately, given the circadian expression profile for MIF in patient plasma, other serum biomarkers may be better-suited to predict ICB response and irAE development, such as soluble CD74 (sCD74), which, to the best of our knowledge, is not known to experience 24 h variability in expression. Further studies on MIF circadian variability will also be necessary to better define how this protein varies in circulation over time.

While serum biomarker measurement is less invasive than tissue or tumor biopsy, fluctuating serum MIF may prove too difficult to use as a reliable biomarker. In this case, it is important to understand whether MIF cycles at the transcript and protein levels in both tumor and healthy tissues, as MIF levels in these tissues could be used as an alternative biomarker of ICB response or irAE development. Tumoral MIF protein expression has been shown to be correlated with plasma MIF cytokine levels in hepatocellular carcinoma [109]. This was not examined across a 24 h period, and time-of-day information is not reported; therefore, it is possible that tumor-derived MIF expression is also circadian. Further work is needed to confirm or refute this possibility. In addition, urine MIF has been used as a biomarker [61], but it is also unknown whether it fluctuates with 24 h rhythmicity.

One of the major limitations of studies examining putative predictive biomarkers is their retrospective nature. Therefore, it is important to design prospective studies that examine CD74 and MIF as biomarkers of ICB response or irAE development across a broad range of cancers, irAEs, and ICB treatments to determine their generalizable use in human subjects [3].

## 8. Conclusions

MIF and CD74 are important molecules in various contexts, including macrophage migration, MHC class II folding, and antigen presentation. However, their roles in solid tumors, ICB treatment, and irAEs have only been investigated more recently. We now know that MIF is expressed by immune, stromal, and tumor cells, and binds to three known non-cognate receptors in addition to CD74, with implications in normal and aberrant immune responses. It has also been shown that MIF and CD74 expression are closely associated with patient outcomes in many types of cancer, and it is widely suggested that both molecules are promising candidate therapeutic targets. Further, both are being investigated as predictive biomarkers of patient response to ICB therapy in various cancers; their utility in this realm is well on its way to being established. On the other hand, relatively few studies have directly reported the use of CD74 or MIF as a predictor of irAE development after ICB treatment. However, given their involvement in the mechanisms of autoimmune disease and their links to the cell types most important in irAE development, it is worth furthering the research on this topic, as well. Further work will undoubtedly shed light on not only the roles of MIF and CD74 in the mechanisms of ICB treatment response and irAE development but also whether they have a place in the clinic as predictive biomarkers.
